# Integrating planetary health education into tertiary curricula: a practical toolbox for implementation

**DOI:** 10.3389/fmed.2024.1437632

**Published:** 2024-10-23

**Authors:** Zerina Lokmic-Tomkins, Liza Barbour, Jessica LeClair, Jeneile Luebke, Sarah L. McGuinness, Vijay S. Limaye, Parvathy Pillai, Maxfield Flynn, Michael A. Kamp, Karin Leder, Jonathan A. Patz

**Affiliations:** ^1^School of Nursing and Midwifery and Health and Climate Initiative, Monash University, Clayton, VIC, Australia; ^2^Division of Planetary Health, School of Public Health and Preventive Medicine and the Health and Climate Initiative, Monash University, Melbourne, VIC, Australia; ^3^Department of Nutrition, Dietetics and Food, Monash University, Clayton, VIC, Australia; ^4^School of Nursing, University of Wisconsin, Madison, WI, United States; ^5^Department of Infectious Diseases, Alfred Hospital, Melbourne, VIC, Australia; ^6^Center for Sustainability and the Global Environment, Nelson Institute for Environmental Studies, University of Wisconsin, Madison, WI, United States; ^7^Department of Population Health Sciences, University of Wisconsin, Madison, WI, United States; ^8^School of Medicine and Public Health, University of Wisconsin, Madison, WI, United States

**Keywords:** planetary health, climate change, environment, education, curriculum development, implementation

## Abstract

**Objective:**

To present a series of case studies from our respective countries and disciplines on approaches to implementing the Planetary Health Education Framework in university health professional education programs, and to propose a curriculum implementation and evaluation toolbox for educators to facilitate the adoption of similar initiatives in their programs. We emphasize the importance of applying an Indigenous lens to curriculum needs assessment, development, implementation, and evaluation.

**Methods:**

Case studies from Australia and United States were collated using a six-stage design-based educational research framework (Focus, Formulation, Contextualization, Definition, Implementation, Evaluation) for teaching planetary health and methods of curriculum evaluation. These components were then mapped to derive the curriculum implementation toolbox reflecting the six-stage design-based educational research framework.

**Results:**

The case studies demonstrated different approaches to successful integration of the Planetary Health Education Framework in medicine, nursing, public health, and allied health disciplines. This integration often involved Indigenous perspectives on environmental stewardship, holistic health, and community well-being into the curriculum. The case studies also highlighted the importance of community engagement, cultural competency, and interdisciplinary collaboration in curriculum development. Findings from case studies were used to propose a curriculum implementation toolbox to assist educators in adapting and integrating planetary health education into their own programs.

**Discussion:**

While valuable frameworks for teaching planetary health in health science programs exist, challenges remain in implementing these frameworks in real-world educational environments. The proposed curriculum implementation toolbox offers practical strategies and resources for educators to incorporate these principles into their teaching. Additionally, the case studies reported here contribute to the growing body of literature on planetary health education pertinent to addressing the triple planetary crisis.

## 1 Introduction

Indigenous Peoples have a deep connection to the natural world, often viewing themselves as an integral part of the environment rather than separate entities. This connection encompasses spiritual, cultural, and practical dimensions, guiding their stewardship practices and sustainable lifestyles through sophisticated knowledge systems emphasizing respect, reciprocity and harmony with nature. In contrast, it is only over the past 60 years that Western science and medicine have directly recognized the close link between our health and the environment. Rachel Carson’s 1962 book, *Silent Spring*, highlighted the impacts of environmental chemicals on songbirds, serving as a wake-up call. In 1987, the United Nations World Commission on Environment and Development published *Our Common Future*—also known as the Brundtland Commission Report—introducing the concept of Sustainable Development (1). The following year, the World Meteorological Organization and United Nations established the Intergovernmental Panel on Climate Change (IPCC) to scientifically assess climate change impacts (2). These events underscore three core challenges we face today—the triple planetary crisis of climate change, pollution and biodiversity loss—each threatening the health of current and future generations (3).

While Earth’s climate naturally changes over time, human activities, particularly the unchecked extraction and burning of fossil fuels, have significantly increased the amount of greenhouse gasses in the atmosphere, driving Earth’s warming. Climate change and pollution are interlinked and their consequences include global temperature shifts; land surface changes; melting ice sheets and sea-level rise; increased intensity and frequency of bushfires/wildfires, heatwaves, storms, floods, and droughts; as well as famine, social disruptions, ecosystem loss and species extinctions (4, 5). There are myriad of long term effects health effects and significant socioeconomic costs (5), often disproportionately affecting Black, Indigenous, low-income, and multispecies communities at the frontlines of climate disasters and industrial pollution (6, 7). Pregnant women, infants and children are also highly susceptible, with resulting reproductive health impairments and adverse pregnancy outcomes (8). Additionally, air pollution can harm cognitive function and increase dementia risk (9).

Biodiversity loss, driven by human activities including deforestation and agriculture, as well as natural disasters such as bushfires, reduces the variety of plant and animal species. This loss leads to crop failures and food insecurity (10), depletes potential sources of medicinal compounds from plants and microorganisms (11), and adversely impacts human physical and mental health (12). Additionally, habitat destruction increases human-animal contact, heightening the risk of zoonotic diseases. This underscores the importance of natural habitat protection (13) and ecological restoration as measures for disease prevention (14).

To promote planetary health, efforts must address the impacts of climate change, pollution and biodiversity loss at community, policy, and systems levels (15). As a carbon emitter and polluter, the healthcare sector must also adopt environmentally sustainable practices, including transitioning to renewable energy sources, revising anesthetic and inhaler protocols and minimizing single-use products and plastics (16). Additionally, the sector must pioneer innovative care models, adopt environmentally sustainable digital health solutions (17), promote reductions in transport-related emissions (16) and provide clinical governance improvements to assure sustainable care delivery. Consumer engagement and prevention investments are key for reducing healthcare demand and facilitating healthcare sector-driven systemic change to promote sustainable and equitable access to healthcare (18).

**Figure 1:**
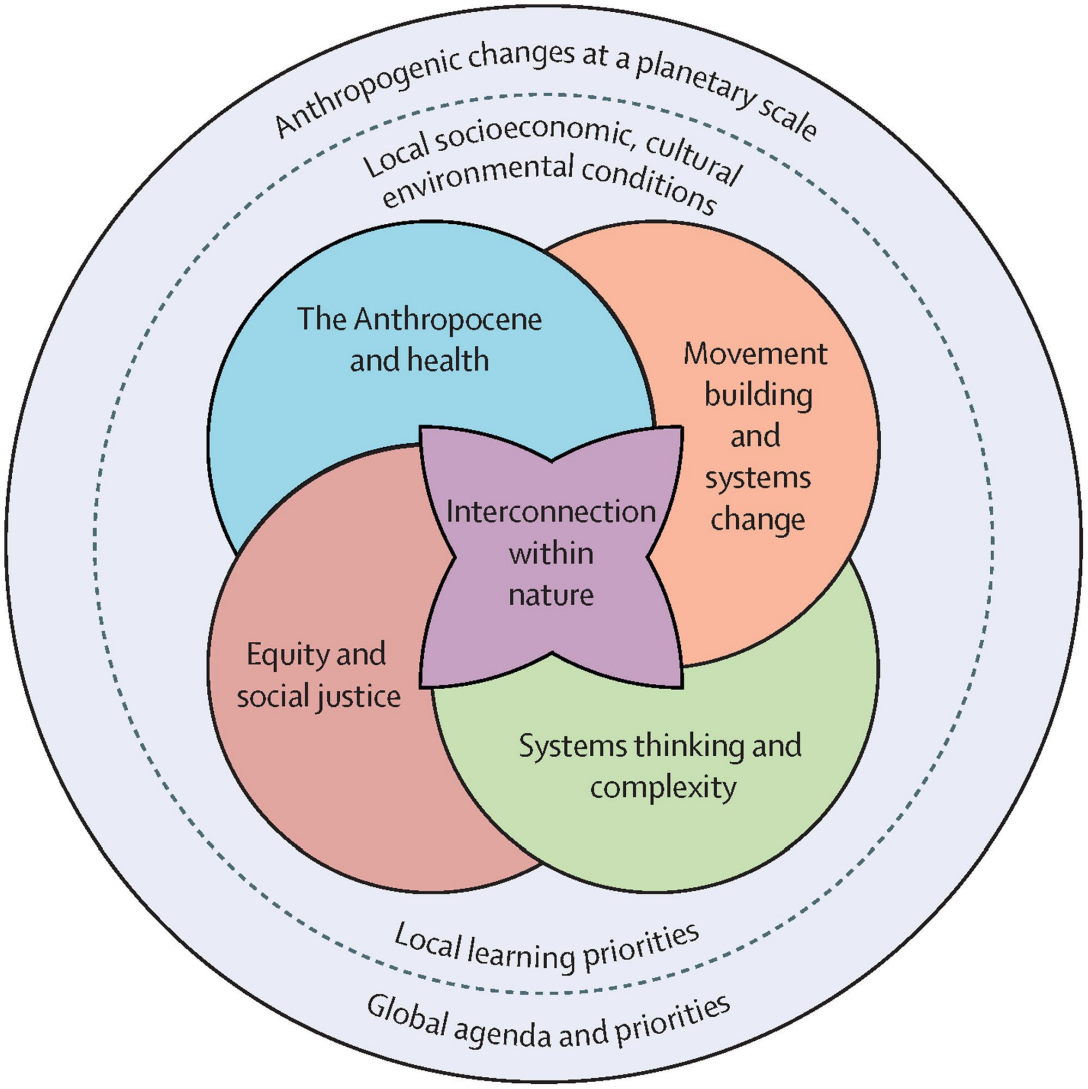
Reproduced from Faerron Guzmán et al. (20) by permission of the authors.

Building healthcare and public health workforce competencies begins with pre-registration education and is strengthened through lifelong learning and mentoring (19). Since the publication of the Planetary Health Education Framework (Figure 1) to address the planetary triple crisis (20), healthcare and public health educators have been working to integrate this framework into their overcrowded curricula. To meet the urgent calls from experts on bold action, wide-spread integration of planetary health education across health disciplines is essential. However, curriculum developers face barriers in mainstreaming planetary health education.

This paper presents a series of case studies from medicine, nursing, public health, and allied health disciplines where the framework has been implemented and offers a toolbox for educators to facilitate similar initiatives in their programs. Rather than attempting to be a comprehensive review of all available curricula, we focus on learnings from our experiences at the University of Wisconsin, Madison in the United States, and Monash University in Melbourne, Australia. By sharing our specific experiences, we aim to provide practical examples of progress, barriers, opportunities and innovations in advancing Planetary Health curricula and education.

### 1.1 Positionality statement

This article was written by scholars who identify as non-Indigenous to Australia (Z.L-T, L.B, S.L.M, K.L), non-Indigenous to the United States (USA; JLeC, V.S.L, P.P, M.F, M.A.K, and J.A.P) and one Indigenous scholar (J.L.), who identifies as an enrolled member of the Bad River Band of Lake Superior Chippewa in Wisconsin, United States.

Jeneile states: “As an Indigenous author, my community and I welcome the collaboration and allyship of non-Indigenous authors, as well as their dedication and commitment to uplifting the voices of the Indigenous community.”

As non-Indigenous authors, we have approached this manuscript with a commitment to allyship, aiming to support and amplify Indigenous voices and knowledge systems. We have undertaken this work from the perspective of non-Indigenous educators and researchers, with a deep dedication to fostering inclusive, culturally responsive, and transformative learning environments. Our collective professional backgrounds in nursing, medicine, public health, and education have shaped our understanding of the complex global challenges addressed by planetary health, particularly at the intersection of environmental sustainability and human well-being.

We further acknowledge that while we each bring different life experiences, positionalities, and biases, we have made every effort to honor Indigenous contributions. This paper reflects our ongoing learning, reciprocity, and commitment to integrating Indigenous knowledge in the effort to address global planetary health challenges.

## 2 Case studies of curriculum development and implementation

### 2.1 Early adopters of planetary health

In 2015, the Rockefeller Foundation–*Lancet* Commission on planetary health released a report that concluded human civilization is mortgaging our future health for current economic growth and development (21). The health of future human civilization depends on thriving natural systems that help provide humans with clean air, clean water, and a buffer against disease outbreaks (21).

Since the report, the planetary health field has grown across the globe and universities are increasingly emphasizing the necessity of using a planetary health lens in education, regardless of the occupation students choose. For example, the London School of Hygiene & Tropical Medicine offers a MSc in Climate Change and Planetary Health (22), and the University of Edinburgh offers a MSc in Planetary Health (23). However, these are only two examples of planetary health education in an area that is growing worldwide.

To serve as a central hub, the Planetary Health Alliance, now led out of Johns Hopkins University in the United States, creates and circulates educational materials and plans annual meetings every 18 months to bring the field together (15). A key educational piece distributed has been the framework on Planetary Health Education (Figure 1) (20).

### 2.2 Medical education

In 2015, leadership from 118 health professions schools worldwide, including the University of Wisconsin School of Medicine and Public Health (UWSMPH), signed the Global Health Educators Climate Commitment, pledging to train the next generation of health professionals to effectively address the health impacts of climate change (24). Since then, there have been multiple calls to action by medical students, faculty, and medical organizations for curriculum on the impacts of climate-related health effects, planetary health and sustainable healthcare (25–29). There are growing examples of how these curricula can and have been incorporated across the continuum of education for medical students to practicing providers (30, 31) and the development of a repository for such curriculum (32). Assessment of planetary health topics have been developed for licensing board examinations for practicing physicians within the United States (personal communication, Cecilia Sorensen 2024), and requirements of the Liaison Committee on Medical Education (the accrediting authority for US medical schools) to address common societal problems, further justify incorporating content on the triple planetary crisis (33, 34).

The UWSMPH MD curriculum now has planetary health-related content in five of the six required preclinical courses that span across the first 18-months of the medical school curriculum. Learning modalities include lectures, small group self-directed learning on asthma and an asynchronous video focused on climate change-related moral injury. There is also a panel discussion featuring Wisconsin tribal community members speaking on food sovereignty, health equity, and the importance of biodiversity and land stewardship. Additionally, students can take a two-week “Climate Change Medicine” elective.

**Figure 2:**
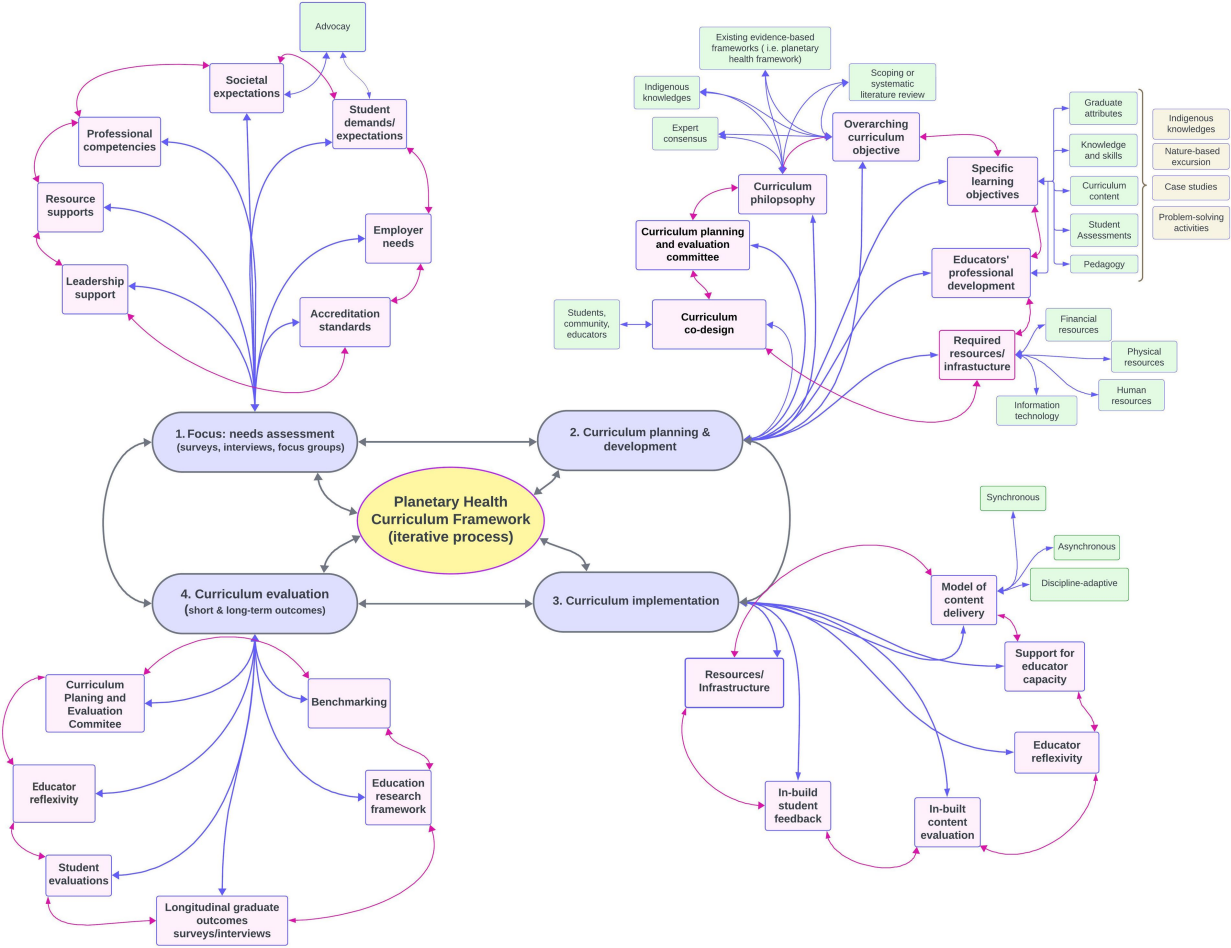
The curriculum development process consists of overlapping, iterative stages (mauve boxes). Each stage functions both as an individual process and as inputs and outputs (gray arrows) for the overall discipline-accommodating planetary health curriculum framework (yellow box). Each subcomponent of the curriculum framework (pink boxes) is interdependent (pink arrows), where the quality of each component affects the quality of the others. Additionally, each building block may require further consideration to meet the specific requirements of its aim (green boxes).

Barriers identified for adding planetary health curriculum include lack of perceived time within the existing curriculum and limited climate change expertise among faculty within UWSMPH. Using existing frameworks (35, 36), a plan has been developed to introduce additional brief asynchronous online videos, rather than time-intensive lectures, and to use these as prework for clinical case-based small-group discussions across preclinical and clinical courses. These efforts will thereby incorporate planetary health considerations into existing pathophysiology-oriented case scenarios.

**Figure 3:**
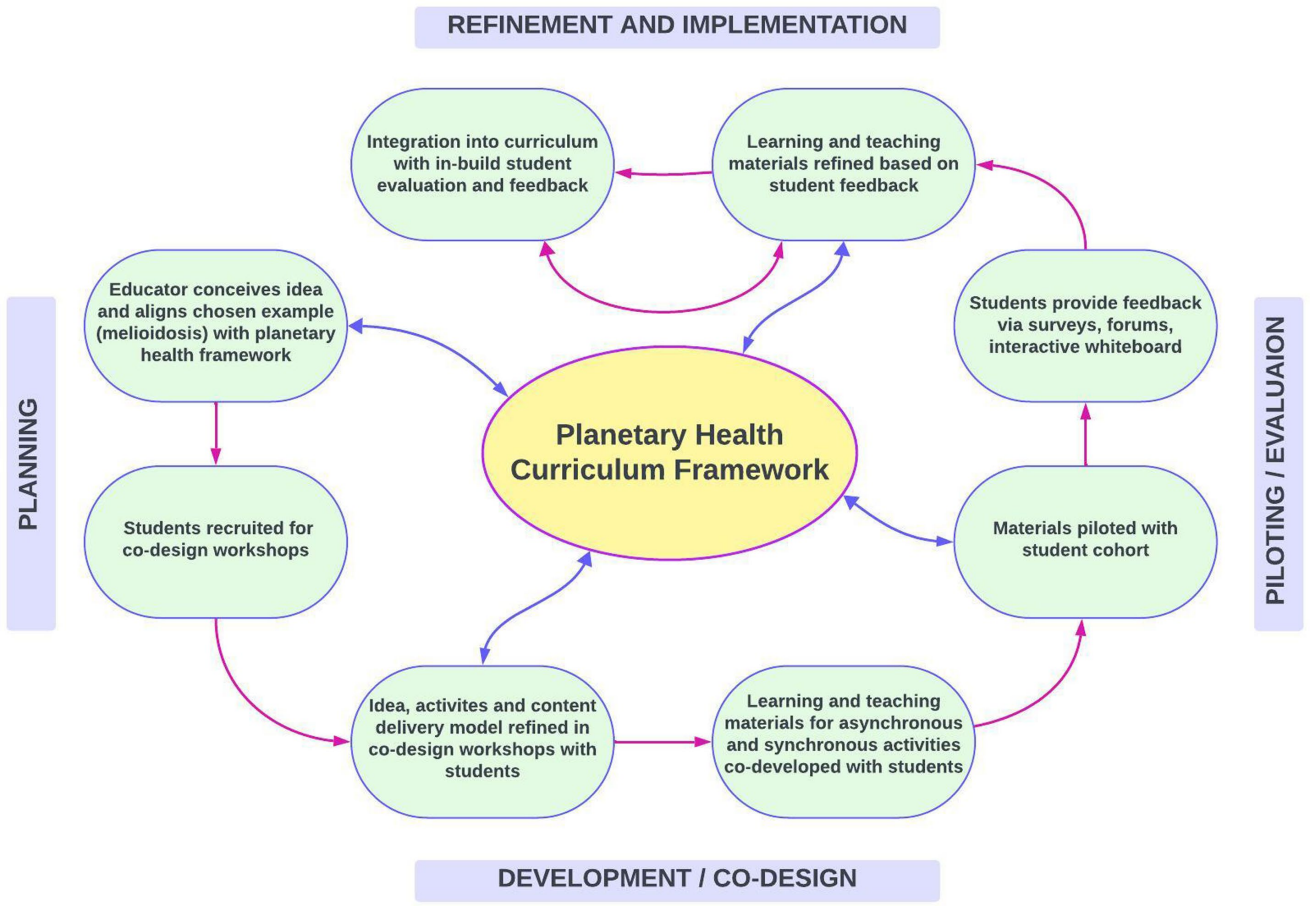
Co-Design and implementation process for a series of learning and teaching activities focused on melioidosis, including asynchronous activities (student-led introductory video on planetary health, drag-and-drop activity illustrating how individual, public and one health concepts relate to planetary health, short lecture on the intersections between melioidosis and planetary health) and a synchronous in-class workshop (based on a hypothetical melioidosis case drafted by students). Materials were piloted with students and refined based on feedback.

### 2.3 Public health education

Black, Indigenous, and other racialized groups in the United States are experiencing worse health impacts from the planetary crisis than white communities (37). To support effective addressing of these health inequities, the American Public Health Association and the U.S. Centers for Disease Control and Prevention’s Climate and Health Program provide technical assistance for local jurisdiction climate planning, identifying and engaging partners, and prioritizing community-driven interventions.

The Global Network for Academic Public Health emphasizes the role of public health schools and programs in planetary health. In the United States, the National *Core Competencies for Public Health Profession*a*ls* direct frontline public health staff to assess community health vulnerability and risks associated with climate change; to communicate environmental impact factors; to collaborate with communities to reduce health inequities by promoting environmental justice; to assess, develop, and implement organizational policies, programs, and services to advance environmental justice. The Association of Schools and Programs of Public Health (ASPPH) has established core competencies for Masters of Public Health (MPH) trainees across five domains (biostatistics, environmental health, epidemiology, health policy and management, and social and behavioral sciences) and seven cross-cutting domains (communications and informatics, diversity and culture, leadership, professionalism, program planning, public health biology, and systems thinking) (38). These core competencies operate as guidelines for accredited schools of public health and a benchmark for non-accredited programs.

Historically, the environmental health domain of MPH training has focused on air and water contamination, drawing on study of well-studied pollutants such as lead, asbestos, and airborne particulate matter. Efforts are underway to enhance core competencies with climate change content through the Climate Change and Health for Public Health Education Toolkit, published in 2022 by the Association of Schools & Programs of Public Health (ASPPH), a US-based organization representing public health schools and programs accredited by the Council on Education for Public Health in the United States and globally (39). Developed with the Global Consortium for Climate and Health Education (GCCHE) (40) this program offers guidance for integrating climate and health content within existing core competency frameworks (35), spanning knowledge and analytical skills, collaboration and communication, policy, public health practice, and clinical practice domains (35). It is of note that GCCHE has developed a suite of free, globally available online courses designed to educate interdisciplinary professionals on the health impacts of climate change and empower them to take action within their fields and communities (41).

At UW-Madison, MPH students typically satisfy their environmental health coursework requirement with a common overview course and engage with planetary health content through elective courses, including health-focused classes on air pollution, climate change, and planetary health (42). The elective courses draw on interdisciplinary approaches and lectures from experts in nursing, environmental studies, chemistry, and zoology from other campus departments. Public health students pursuing MS and PhD degrees also take these electives and integrate planetary health training into their research training through joint, double, and dual degree programs (43) which facilitate interdisciplinary training and research experiences but can require additional course-loads. They enhance student fluency and covering areas such as biodiversity threats to disease spillover risks (44), extreme heat (45), and indoor air pollution (46).

### 2.4 Nursing education

Recognizing the growing climate crisis, the International Council of Nurses (ICN) has integrated climate change into its Code of Ethics (47), and, along with the American Nurses Association (ANA) Code of Ethics, inspires nurse action and advocacy for environmental preservation (48). The ANA recently issued a statement on nursing’s responsibility in addressing climate change and climate justice, urging integration of climate and health science into nursing education (49), and also updated the scope and standards of public health nursing to include environmental and planetary health and environmental justice (50). The National Academies of Sciences, Engineering, and Medicine’s Future of Nursing 2020–2023 report highlights the intersection of environmental health, racism, discrimination, planetary disasters, and implications for nursing (51).

Growing support exists for integrating planetary health into nursing curricula. For example, the Nurses Climate Challenge, a collaboration between the Alliance of Nurses for Healthy Environments and Health Care Without Harm, aims to educate nurses about climate change, partnering with nursing schools to embed climate change content into curricula from baccalaureate to doctoral programs (52). It shares educational and advocacy materials on climate change impacts for nurses to use in educating colleagues and patients. Nurse scholars have proposed adding planetary health as the ninth concept in the American Association of Colleges of Nursing (AACN) Essentials competency-based education framework (53). While the AACN has not adopted this yet (54), following Flatten et al. (53), the UW-Madison School of Nursing plans to integrate planetary health as a concept that, like diversity, equity, and inclusion, will be woven across AACN Essentials domains and competencies. The Planetary Health Education Framework is successfully applied to support the implementation of the AACN Essentials in nursing education (53). The Nursing Planetary Health Report Card is another international transdisciplinary student-led initiative to assess schools’ work in planetary health and suggest improvements (55).

The University of Wisconsin (UW)-Madison School of Nursing offers undergraduate, Doctor of Nursing Practice (DNP), and PhD programs, integrating planetary health topics across all programs since 2018. Faculty utilize exemplars to demonstrate how nursing concepts can be applied to multiple issues. The Social Ecological Model is taught across subjects to demonstrate nursing strategies for addressing planetary health at various levels. Students engage in readings and a lecture on communication strategies related to planetary health and climate justice, and collaborate to devise pitches for implementing the Nurses’ Climate Challenge initiative in clinical sites or future workplaces. Classroom sessions often feature guest speakers, including sustainability experts from local health systems, enhancing student learning.

The Australian Nursing and Midwifery Accreditation Council (ANMAC) is the accreditation authority responsible for accrediting education providers and programs of study for the nursing and midwifery profession in Australia (56). Currently under review, the Registered Nurse Accreditation Standards (2019) outlines in *Standard 3: Program of Study* that *‘Teaching and learning reflects contemporary practices in nursing, health and education, and responds to emerging trends based on research, technology and other forms of evidence*.’(Standard 3.4) and that the program’s content and subject learning outcomes ensure ‘*integrated knowledge of regional, national and global health priorities, including mental health and care of the older person’* (Standard 3.5 b) (57). Thus, to meet Standard 3.5b, nursing preregistration programs must demonstrate the integration of contemporary issues impacting the nursing profession, such as the United Nations Sustainable Development Goals (58). In response, the Planetary Health in Nursing & Midwifery – Research & Education Collaborative produced a planetary health curriculum framework, inclusive of Indigenous knowledges, outlining key knowledge and skills with a specific focus on climate change (59). Disseminated via the Council of Deans of Nursing and Midwifery, this framework helps Australian nursing schools meet ANMAC’s Standards 3.4 and 3.5b on planetary health. The next challenge is to embed the planetary health framework into the upcoming accreditation standards, an essential step toward equipping the entire future Australian nursing workforce to engage and lead in planetary health.

### 2.5 Centering Indigenous voices in the promotion of planetary health in nursing

Indigenous-specific knowledge and planetary health have been integrated into the nursing curriculum at the UW-Madison School of Nursing through our annual Native Nations Nursing, Helpers, and Healers (NNNHHS) summit, held each fall. The summit has been designed and refined over the past 10 years to strengthen community-academic partnerships between the University and the 12 sovereign tribal nations in Wisconsin. The NNNHHS summit emphasizes the collaborative efforts necessary between Indigenous communities, tribal leaders, healthcare providers from both tribal and non-tribal entities, community members, and researchers to deliver effective health programs tailored to promote the health and wellbeing of Indigenous peoples and their communities. The summit also brings future healers, faculty, staff, and community members as a learning and healing community. Due to the continuation of colonial influences and systems, there are limitations in exposure and opportunities to learn about Indigenous ways of knowing and being. Therefore, the NNNHHS summits provide a safe place for students, faculty, healers, and community members to come together and learn from one another. Blending Western sciences with traditional Indigenous knowledge and values to best meet Indigenous patients’ needs is important, as is centering planetary health and justice in our work.

There has also been a purposeful integration of planetary health within the undergraduate and graduate nursing curriculum, with incorporation of several modules emphasizing the intersection of environment and health and the impact of climate change on the health and wellbeing of individuals, families, and communities. There is also an emphasis on historically excluded populations, including Indigenous communities in North America and globally, which serves as a step toward decolonization of the curriculum.

Indigenous peoples hold deep generational knowledge of their environments and sustainable practices that can significantly contribute to addressing climate change and promoting planetary health (60, 61). They also have vast experience managing delicate ecosystems through sustainable practices that have been honed and passed on for centuries. Indigenous perspectives can offer invaluable lessons critical to addressing climate justice and promoting planetary health (60, 61), and providing insights as the protectors and stewards of their lands (60).

Many Indigenous Peoples and communities have associated human-caused climate change and land degradation with femicide against our Mother Earth, a powerful concept that underscores the severity of human-caused climate change as well as ongoing land degradation and destruction. It also highlights the interconnectedness and intersectionality of environmental destruction and violence against Indigenous women, drawing attention to how both result from systems of exploitation and disrespect for life in all forms. Equating climate injustice with femicide is an urgent call to action to recognize and rectify the harm we are causing the planet (60), and recognizes respect of the feminine and Indigenous health and land tenure rights as interconnected determinants of planetary health (60). Indigenous communities often have traditional laws and practices that inherently respect the natural world, and tribal nations have been the leaders in implementing Rights of Nature legislation within their sovereign territories (60). These perspectives are increasingly being recognized and integrated into modern legal frameworks by implementing “rights of nature” laws, for which Indigenous peoples’ traditional ecological knowledge can be a valuable foundation.

### 2.6 Allied health education

Dietitians can help transform two key systems contributing to climate change, pollution, and biodiversity loss: the food system and the healthcare system. The current food system contributes a third of global greenhouse gas emissions, while driving diet-related disease and biodiversity loss, with dominant agricultural practices threatening 86% of species at risk of extinction (62, 63).

In Australia, recently updated dietetic competency standards contain greater emphasis on planetary health (64, 65). The Code of Conduct also requires dietitians to “participate in efforts to support progress toward sustainable food production, food systems and food and nutrition security for all, where possible” (66). Monash University offers both undergraduate and postgraduate degrees (Bachelor of Nutrition Science and Masters of Nutrition and Dietetics). However, current accreditation standards lack adequate planetary health curricula to prepare the future nutrition and dietetics workforce to address the triple planetary crisis. Therefore, Monash University educators and program directors are proactively integrating planetary health education into their curricula.

The approach has differed for each program (Table 1). The undergraduate program includes a dedicated unit on food sustainability systems, while the postgraduate program integrates a ‘food systems’ lens throughout the curriculum. This integration allows students to engage with planetary health education throughout their classes, placements, research and assessments. Monash is not the first to apply a food systems lens to their dietetic programs (67, 68), and future research will examine the impact of this approach on learner experiences and graduate attributes.

**Table 1:** Overview of planetary health curricula in nutrition and dietetics programs offered at Monash University.

Program	Bachelor of nutrition science	Masters of nutrition and dietetics
*Approach to embedding planetary health curricula*	A stand-alone, compulsory unit ‘Food Sustainability Systems’ was developed and first delivered in 2016. The unit is facilitated by two educators with expertise in planetary health.	A 2022 curriculum review led to embedding a ‘food systems approach’ to the whole dietetics program. Educators with varying levels of expertise in planetary health facilitate this curriculum across multiple units.
*Description of planetary health curricula*	Learners complete this 12-week unit, with a 3-h in-person workshop each week in addition to self-directed pre-class (~2 h) and post-class (~1 h) activities.	Learners complete a dedicated unit ‘Food systems for nutrition and dietetic practice’ and complete food systems-oriented activities during their clinical and public health placements.
	Prioritization is given to pre-, in-and post-class activities that expose learners to Indigenous peoples’ knowledge about food systems.	
*Learning and teaching examples*	Assessment tasks: Learners conduct a comprehensive Food System Audit of their chosen local government area and present an oral Counselor Brief, with prioritized, evidence-based policy actions to improve planetary health outcomes. Field trips: (i) Local urban agriculture site supplying a state-wide food relief charity, and (ii) Foodbank for people seeking Asylum in Australia, exemplifying dignified and inclusive emergency food relief.	Workshop activities: Learners are introduced to the key drivers, prevailing power relations within food systems, and the role of dietitians in navigating these in various ‘micro food systems’ to promote planetary health and equity. Field trips: (i) Facilitated workshop on soil health, composting and urban agriculture at an urban farm and social enterprise hub, and (ii) Guided tour of an on-campus residential food service focused on reducing food waste, using seasonal and local produce and connecting with on-site fruit, vegetable and herb gardens.

In both programs a ‘Public Health Nutrition’ unit offers a workshop co-designed by educators and students. The workshop emphasizes values-based practice, focusing on reciprocity and respect that are critical to Indigenous peoples’ practices to maintain sustainable food systems in Australia for over 65,000 years (69). This co-designed curricula, part of a Faculty-wide fellowship project, will be shared with educators from other disciplines to support the integration of more planetary health content in their programs.

### 2.7 Interdisciplinary education

While current accreditation standards do not require planetary health curricula, the Monash’s Master of Public Health Program introduced a “Global and Planetary Health” stream in 2023. As part of a faculty initiative, educators were encouraged to become “planetary health education champions.” In this context, students and educators created interconnected learning activities, an example of which was on melioidosis for the Infectious Diseases Epidemiology unit. Figure 3 outlines the co-design and implementation process, designed to facilitate student input on content and delivery to enhance the learning experience.

**Figure 4:**
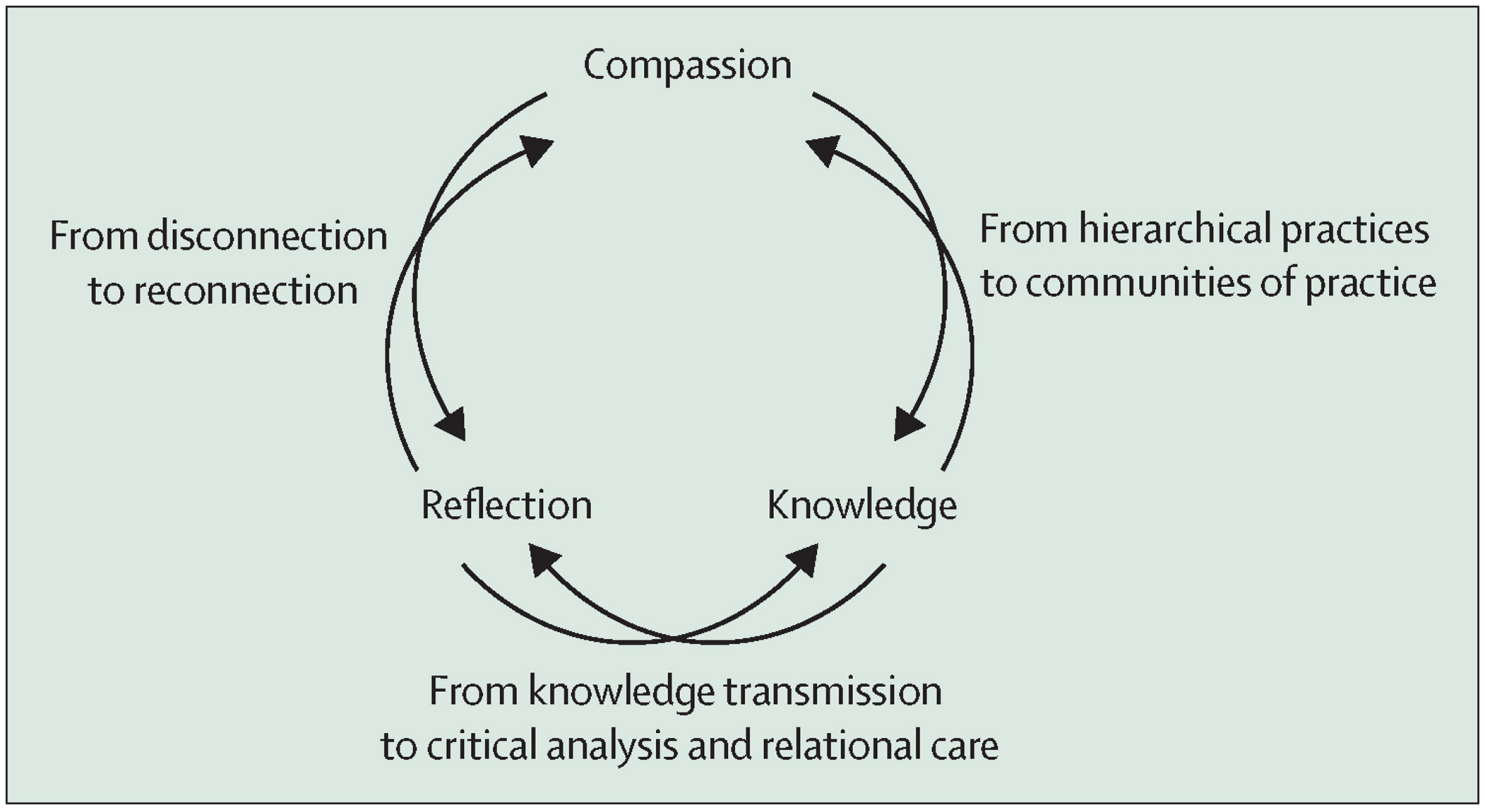
Reproduced from Redvers et al. (76) by permission of the authors.

Melioidosis, caused by the bacterium *Burkholderia pseudomallei*, is an infectious disease affecting humans and animals, particularly in Southeast Asia and northern Australia. It highlights the connection between planetary health and infectious diseases, reflecting key aspects of the Planetary Health Education Framework (Table 2) (20). This includes illustrating how climate change and ecosystem disruptions affect disease risk and epidemiology, and showcasing equity issues in the uneven distribution of environmental and health impacts. Despite its global importance, melioidosis remains under-recognized, perpetuating existing inequities (70).

**Table 2:** Alignment of melioidosis case study with planetary health education framework and key planetary health concepts.

Planetary health education framework domain	Key concept	Alignment with example of melioidosis
Anthropocene and health	Climate change	Increased rainfall and temperature linked to higher melioidosis risk (89) Geographic expansion of melioidosis during La Niña events (90)
	Land-use change and ecosystem disruptions	Urban expansion and construction associated with melioidosis cases (91)
	Globalization and other human activities	Melioidosis spread to non-endemic areas through commercial products (92) and pet animals (93)
Systems thinking	Dynamic interactions among complex systems	Complex interactions between risk factors (e.g., diabetes), socio-economic conditions, healthcare system capacity and disease risk and outcomes
Equity and social justice	Uneven distribution of disease impacts	Disproportionate impact on rural poor populations in low-and middle-income countries (70) Variation in mortality rates reflecting differences in healthcare access and risk factor prevalence (70)
Movement building and systems change	Advocacy	Calls to recognize melioidosis as a neglected tropical disease for increased awareness and funding (70)

## 3 Curriculum implementation toolbox

Regardless of the discipline where the planetary health education curriculum framework has been implemented, there are common principles that can be derived from each of the above case studies. These common principles are summarized in Figure 2 and encompass curriculum needs assessment, design and development, implementation and evaluation.

### 3.1 Curriculum needs assessment

The call for curriculum change reflects society’s demand for health professionals to deliver safe, quality healthcare. These demands are often reflected in accreditation requirements and associated competencies although sometimes these lag behind societal norms. To assess the need for such change and to bring the project into focus (71), a curriculum needs assessment can evaluate the current state and identify what to teach within the human health-environment connections. There are three components to consider: (1) What are the needs of target learners; (2) What is the goal of the curriculum; and (3) in which context will this curriculum be delivered? Required data can be gathered through surveys, interviews, focus groups, auditing the existing curriculum and engaging key partners (employers, students, experts, etc.,) to understand student needs, employer needs, accreditation standards and relevant competencies. These data are also essential for leadership buy-in as their support enables required resources to be made available for curriculum development, implementation and evaluation (72). Regardless of discipline, some common principles apply to embedding planetary health education in the curriculum, such as top-down approaches driven by accreditation and regulatory requirements, and bottom-up approaches led by students and educators. Engaging various stakeholders, including Indigenous Peoples, community members, healthcare providers, policymakers, educators, and students, is essential in curriculum needs assessment.

To assess learner needs, the first question to ask is: Who are our learners? What would learners want to know or achieve in the context of planetary health education? This can be assessed by involving as many relevant key players as can be identified. Including healthcare providers, policymakers, educators, and students as key players also helps and can be achieved by developing a Curriculum Planning and Evaluation Committee. Assessing prior knowledge and current attitudes toward environmental issues and their personal connection to the natural world, can be leveraged to create a scaffolded curriculum. Such data can inform strategies for fostering environmental awareness and responsibility and establishes a baseline understanding of concepts like climate change and biodiversity, aiding in measuring curriculum impact. Considering student engagement in the digital age and potential impacts of generative artificial intelligence on assessment components are key strategies to ensure authentic learning (73).

### 3.2 Curriculum planning and development

To contextualize the pedagogy, the process of developing clear and measurable *learning objectives, activities, assessments and resources* for the planetary health curriculum starts with reviewing and adapting the Planetary Health Education Framework for health science education (20) to reflect local needs. The learning outcomes must encompass knowledge, skills, and dispositions necessary to understand and address planetary health challenges, in particular the triple crises of climate change, pollution and biodiversity loss. National professional accreditation standards exist to ensure the curriculum aligns with their expectations, and these can be further extended through UNESCO’s Education for Sustainable Development competencies (74). The curriculum philosophy, which will provide a set of common values and principles to guide all subsequent decision-making when developing, evaluating and refining the curriculum, can be developed in collaboration with the Curriculum Planning and Evaluation Committee (75).

To develop necessary knowledge, skills and graduate attributes, the pedagogical strategies and the curriculum must be tailored to geographical context and locally relevant climate challenges, prioritizing key topics and concepts resonating with the student’s sense of belonging to the place. Structural reforms may be necessary in how diverse knowledges are produced and disseminated (76). To ensure a comprehensive exploration of human-environment interconnectedness, use realistic case studies to integrate climate change, water quality, biodiversity loss, food systems, and sustainability for culturally appropriate and locally relevant action decisions (77). The Planetary Health Alliance provides a suite of case studies for this purpose (78). Balancing of content depth with breadth across various planetary health issues, while fostering hope and empowering students to act, is required.

The Planetary Health Alliance defines planetary health as a solutions-oriented, transdisciplinary field and social movement focused on analyzing and addressing the impacts of human disruptions to Earth’s natural systems on human health and all life on Earth’ (15). A transdisciplinary approach involves integrating knowledge and methods from various disciplines, including non-academic perspectives, to address complex real-world problems. This approach is crucial for implementing practical solutions in real-world settings because it brings together diverse stakeholders, including policymakers, practitioners, and community members, ensuring that solutions are holistic and applicable in practice (79). While a transdisciplinary approach is essential for implementing practical solutions in real-world settings, the core of planetary health education lies in interdisciplinary connections. This refers to the collaboration and integration of insights from different academic disciplines to understand and address the complex challenges of planetary health. In education, the emphasis is on fostering interdisciplinary connections because they enable students to develop a comprehensive understanding of planetary health issues, considering their biological, environmental, social, and economic dimensions (80). This broad-based understanding is crucial for identifying and analyzing problems before they can be addressed through transdisciplinary action (81).

To reproduce this in the classroom, encourage diversity by bringing together students from different backgrounds such as social sciences, language arts, agronomy, environmental studies, engineering, or mathematics, alongside those in healthcare, public health, and veterinary medicine. Explore ways to infuse planetary health concepts into various subject areas for a holistic understanding. However, evidence suggests that health professions educators have limited capacity and confidence to teach planetary health (82), likely due to emerging nature of this field and their lack of formal training. Involving students as partners in curriculum development, using the principles of co-design, offers a practical approach to increase student engagement and educator capacity (83), as outlined in the case of melioidosis (Figure 3).

Perhaps the most challenging aspect of any curriculum design is creating authentic assessments, particularly those that bring a group of diverse individuals together. Developing formative and summative assessments to gauge student learning and the effectiveness of the curriculum while reflecting genuine engagement with the planet is difficult. Usually, formative assessments involve quizzes, discussions, or self-reflection activities; our suggestion is to replace these activities by involving nature into the teaching and assessment tapestry. Using longitudinal case studies reflecting real-world environments as highlighted above, students can be grounded in experiential, scaffolded learning about the interconnectedness of nature and human health. For summative assessments which traditionally include projects, presentations, or essays, consider expanding these to meaningful engagements with real community needs, such as Indigenous Peoples health needs and Indigenous knowledges. Students can be taught to critically evaluate data to develop innovative solutions to environmental challenges (77). While technology can enhance learning and engagement with planetary health topics, it can also alienate us from the natural world (84), so where technology is used, for example simulations, online databases, or interactive presentations, consider how can these support citizen science projects that focus on planetary health (85). Another consideration is managing and enhancing learners’ tolerance of uncertainty. The climate crisis is filled with uncertainty, and educators must nurture favorable responses to uncertainty in the way they develop and implement planetary health education. This can be achieved through strategic use of stimulus and moderators, as explained by Lazarus and Stephens in their handbook for educators, which includes discipline-specific examples of planetary health education (86).

### 3.3 Curriculum implementation

To implement planetary health into the curriculum, decisions must be made regarding when and where in the program this integration should occur. If a single subject is shared across disciplines, each discipline should have the freedom to implement the subject in a manner that aligns with their professional needs. During pilot implementation and regardless of whether the mode of curriculum delivery is asynchronous or synchronous, virtual, hybrid or face-to-face, it is of value to build in evaluation processes that will run alongside the curriculum delivery. These evaluation processes can include learning platform analytics, built-in student feedback via surveys or open invitation to make constructive comments either anonymously or otherwise and setting up mechanisms for long-term follow ups on student career outcomes. It also requires teaching staff to reflect on the implementation process to decide on necessary adjustments to improve outcomes.

### 3.4 Curriculum evaluation

The process for curriculum evaluation should be established at the project’s inception using education research-based frameworks such as Kirkpatrick-Barr Framework (87). This approach allows for detailed, prospective data collection during the curriculum’s implementation, as well as longitudinal data on student outcomes and career choices, helping to refine curriculum content and delivery. The Curriculum Planning and Evaluation Committee can review these results and act as a source of further direction on curriculum development. Another opportunity for evaluation is curriculum benchmarking against set standards that can be developed in collaboration with other universities (88). Revised curriculum can then be implemented and reviewed on a regular basis. To achieve real transformation, a praxis for planetary health education has been proposed, which is influenced by Indigenous knowledge systems, critical theory, and an ethics of care (76) (Figure 4). This praxis is a convergence of compassion-knowledge-reflection that best enables the formation of planetary stewards. Utilizing this praxis when evaluating the implementation of planetary health education is both a process and outcome that advances social and environmental justice (76).

## 4 Conclusion

Integrating the Planetary Health Education Framework into health professional degrees is essential for addressing the interconnected challenges of human health and the triple planetary crisis of climate change, biodiversity loss and pollution. The work presented here draws on educators’ experiences in overcoming barriers to equip future healthcare professionals with the knowledge and skills to reduce environmental health impacts and promote sustainable healthy lifestyles. Co-designing curriculum between students and educators shows great promise as an approach to increase both student engagement and educator capacity to facilitate planetary health education. To date, this often has been driven by planetary health champions, but top-down accreditation standards and bottom-up student demand for curriculum is accelerating. One limit to our work stems from the preponderance of literature reflecting experiences from the high-income counties. Yet, planetary health programs are growing globally, and new lessons will inevitably emerge from low-to-middle-income settings.

Incorporating an Indigenous lens enriches our proposed toolbox, as Indigenous knowledge systems provide critical insights into sustainable living and the interconnection of people and ecosystems. By learning from Indigenous perspectives, health professionals can develop culturally safe approaches that respect and incorporate Traditional Ecological Knowledges. However, it is in high-income countries that such integration needs improvement.

In summary, the proposed implementation toolbox must evolve with new perspectives, ever-advancing technology, and increasing resources considering the urgency of the triple planetary crisis. We offer a starting point for practical strategies and resources, ensuring systematic and iterative curriculum development, and allowing for continuous improvement and adaptation to changing needs for incorporating the planetary health into mainstream health education.

## Data Availability

The original contributions presented in the study are included in the article/supplementary material, further inquiries can be directed to the corresponding author.
